# Nurse-led preprocedural Valsalva maneuver training with a maximal expiratory pressure device during transesophageal echocardiography in South Korea: a quasi-experimental study

**DOI:** 10.7586/jkbns.26.014

**Published:** 2026-08-27

**Authors:** Kyoungmin Kim, Yoonju Lee, Sanghyun Lee

**Affiliations:** 1 College of Nursing, Pusan National University, Yangsan, Korea; 2 Cardiovascular Center, Pusan National University Yangsan Hospital, Yangsan, Korea

**Keywords:** Echocardiography, transesophageal, Valsalva maneuver, Maximal respiratory pressures, Breathing exercises

## Abstract

**Purpose:**

This study examined the effects of preprocedural Valsalva maneuver (VM) training using a maximal expiratory pressure (MEP) device on VM performance and patient discomfort during transesophageal echocardiography (TEE).

**Methods:**

This study used a time-lagged, posttest-only, nonequivalent control group quasi-experimental design. A total of 129 patients undergoing TEE were included in the final analysis, including 67 in the control group and 62 in the intervention group. The control group received written educational materials and standardized verbal instructions, whereas the intervention group received preprocedural VM training with real-time feedback using an MEP device. Analysis of covariance was additionally performed to adjust for potential confounders, including age, sex, education level, and previous endoscopy experience.

**Results:**

The number of attempts required for successful VM was significantly lower in the intervention group than in the control group (F = 45.07, *p* < .001). The VM success rate was higher in the intervention group (F = 51.52, *p* < .001), and interatrial septal excursion was greater in the intervention group (F = 32.46, *p* < .001). Nurse-observed discomfort was significantly lower in the intervention group (F = 21.73, *p* < .001), whereas patient-reported discomfort did not differ significantly between groups (F = 0.15, *p* = .701).

**Conclusion:**

Nurse-led preprocedural VM training using an MEP device may promote more effective VM performance and help reduce observable discomfort during TEE. These findings suggest that preprocedural VM training with real-time feedback may enhance patient cooperation and improve procedural efficiency.

**Trial Registration:**

Clinical Research Information Service (CRIS), KCT0008944.

## INTRODUCTION

### 1. Background

Effective patient cooperation is essential for the smooth performance of transesophageal echocardiography (TEE). Nurses play a key role in preparing and guiding patients; however, bedside instruction for the Valsalva maneuver (VM) during TEE remains unstandardised. The VM is crucial for detecting patent foramen ovale (PFO) [1], for which TEE is the reference standard for anatomical characterisation [2]. PFO, present in 20%–25% of adults, is a major cause of cryptogenic stroke, highlighting the importance of accurate and reproducible assessment [3,4]. Upon release of the VM, right atrial pressure transiently exceeds left atrial pressure, facilitating right-to-left shunting through a PFO [5]. This is accompanied by leftward displacement of the interatrial septum, which is used clinically to assess the adequacy of VM performance [6]. In this context, interatrial septal excursion (IASE) may also reflect the quality of VM performance and can be considered a supportive indicator of maneuver adequacy [7]. However, probe-related discomfort, gag reflex, and anxiety may interfere with VM performance [8,9]. Inadequate VM often requires repeated attempts, leading to prolonged examination time and increased patient discomfort.

Various techniques, such as coughing, abdominal compression, and voluntary straining, have been used to assist VM performance; however, these methods remain poorly standardised and are limited by insufficient pressure generation, operator dependency, and lack of feedback after probe insertion [10,11]. Traditional bedside assessments, such as abdominal palpation, are also subjective and lack reproducibility [12]. Moreover, TEE presents greater discomfort and reduced patient cooperation compared with transthoracic echocardiography and lacks a standardised approach to preprocedural training [13].

Maximal expiratory pressure (MEP) devices may be used to address these limitations. Oral pressure measured using an MEP device is generated during forced expiration against a closed glottis and is closely associated with an increase in intrathoracic pressure [14]. This mechanism reflects the physiological basis of the VM, in which intrathoracic pressure is increased through expiratory effort against a closed airway [15]. Accordingly, oral pressure measured using an MEP device can serve as an indirect indicator of the intrathoracic pressure required for effective VM performance [16]. In contrast to conventional education, which relies on subjective assessment by nurses (e.g., abdominal palpation) and patient performance based on verbal instruction, MEP device-based training enables both patients and nurses to monitor target pressure and duration via a display [17].

In the context of TEE, this allows patients to practice generating sufficient pressure prior to probe insertion, thereby facilitating adequate maneuver performance. In addition, MEP devices provide real-time visual feedback, enabling patients to monitor their performance and improve reproducibility. These devices have been widely used in respiratory assessment and rehabilitation, demonstrating safety, objectivity, and reproducibility [18]. Thus, they may be useful for standardised preprocedural VM training.

Preprocedural education in various clinical settings has been shown to reduce anxiety, improve patient cooperation, and enhance procedural outcomes [19]. Structured instruction prior to endoscopic or radiologic examinations has also been associated with increased patient satisfaction and improved procedural efficiency [20]. Nurses are well positioned to provide such education and deliver individualised VM training.

Accordingly, nurse-led, MEP-based VM training may represent a feasible and cost-effective strategy to improve patient cooperation, reduce repeated attempts, and alleviate discomfort during TEE. This study aimed to evaluate the effects of nurse-led preprocedural VM training using an MEP device on the number of attempts required to achieve a successful VM, VM success rate, and patient discomfort during TEE.

### 2. Study aim

The purpose of this study was to evaluate the effects of preprocedural VM training using an MEP device on VM performance, including the number of attempts required to achieve a successful VM, VM success rate, and interatrial septal excursion, and on patient discomfort, including nurse-observed and patient-reported discomfort, during TEE.

### 3. Hypothesis

The hypotheses of this study were as follows:

**Hypothesis 1.** The intervention group that received preprocedural VM training using a MEP device would require fewer attempts to achieve a successful VM than the control group.

**Hypothesis 2.** The intervention group that received preprocedural VM training using an MEP device would show a higher VM success rate than the control group.

**Hypothesis 3.** The intervention group that received preprocedural VM training using an MEP device would show greater IASE than the control group.

**Hypothesis 4.** The intervention group that received preprocedural VM training using an MEP device would show lower patient discomfort than the control group.

**Sub-hypothesis 4-1.** The intervention group would show lower nurse-observed discomfort than the control group.

**Sub-hypothesis 4-2.** The intervention group would show lower patient-reported discomfort than the control group.

## METHODS

### 1. Study design

This quasi-experimental study used a posttest-only, non-equivalent control group design to evaluate the effects of nurse-led preprocedural VM training using a MEP device before TEE. To minimize cross-group contamination, participants were sequentially enrolled using a time-lagged design, with the control group recruited first, followed by the intervention group under the same standardized procedures. This study was reported in accordance with the Transparent Reporting of Evaluations with Nonrandomized Designs (TREND) statement.

### 2. Participants

The study was conducted at Pusan National University Yangsan Hospital, a tertiary university-affiliated medical centre in South Korea. Eligible participants were those scheduled for TEE who met the following criteria: (1) aged 18–80 years, (2) no prior experience with TEE, (3) able to communicate effectively, and (4) provided written informed consent.

Exclusion criteria included: (1) contraindications to the VM as determined by a physician [21], (2) diagnosis of atrial septal defect or prior atrial septal defect closure, (3) receipt of preprocedural sedation, (4) inability to maintain the left lateral decubitus position due to physical limitations (e.g., fracture), and (5) impaired consciousness or inability to make decisions.

Sample size was calculated using G*Power 3.1 based on an independent t-test (two-tailed) with a significance level of α = .05, power = .80, and a medium effect size (Cohen’s d = 0.5), yielding a required sample size of 64 participants per group [22]. Considering the dropout rates reported in previous studies [23], a 10% dropout rate was applied, resulting in a target sample size of 70 participants per group.

**Figure 1.**
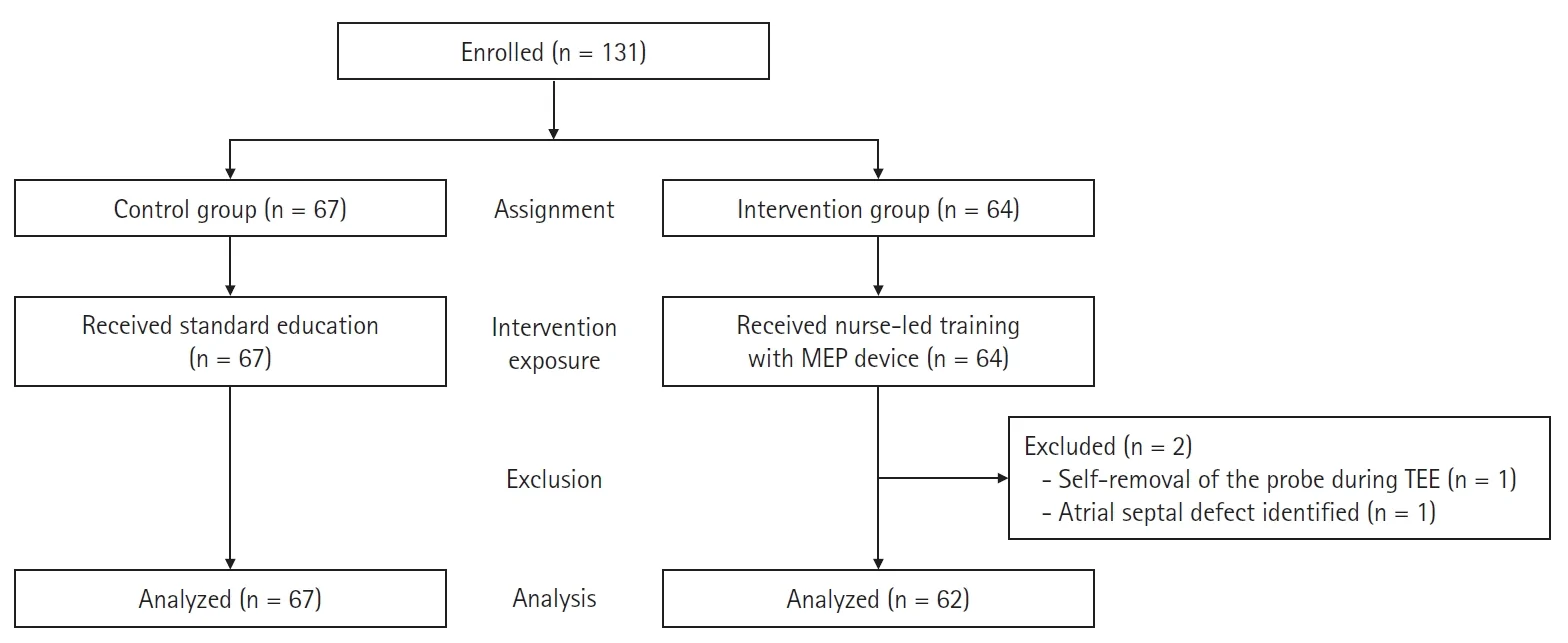
Participant flow through the study. MEP = Maximal expiratory pressure; TEE = Transesophageal echocardiography.

Due to institutional scheduling constraints, recruitment concluded earlier than planned; however, a total of 129 participants (control n = 67; intervention n = 62) were included in the final analysis. The flow of participants through each stage of the study is presented in Figure 1.

### 3. Measurements

The outcome variables of this study were VM performance, assessed by the number of attempts required to achieve a successful VM, individual-level VM success rate, and interatrial septal excursion, and patient discomfort, assessed by nurse observation and patient self-report. General characteristics, including age, sex, education level, occupation, marital status, and previous endoscopy experience, were assessed before the procedure using a structured questionnaire. These variables were collected as baseline characteristics because they could be associated with patients’ ability to understand and perform the VM, cooperation during TEE, or procedure-related discomfort.

#### 1) Number of VM attempts

The number of attempts required to achieve a successful VM was defined as the number of attempts made until the first adequate VM performance during TEE, as determined by an experienced cardiologist [24]. This variable indicates how many attempts were needed for a patient to perform an adequate VM during the examination; therefore, fewer attempts suggest that the maneuver was achieved more easily and with less need for repeated instruction. The number of attempts was limited to a maximum of five [25]. A successful VM attempt was determined by the cardiologist as a binary clinical judgement based on whether adequate leftward displacement of the interatrial septum was observed immediately after VM release during TEE [26]. The same cardiologist applied this criterion consistently to all participants.

#### 2) VM success rate

The individual-level VM success rate was operationally defined as the proportion of successful VM attempts among the total number of VM attempts performed by each participant during TEE. Because VM attempts were performed until an adequate VM was achieved or until the maximum of five attempts was reached, this variable was interpreted as a complementary attempt-based indicator of VM performance rather than as an outcome fully independent of the number of attempts. The individual-level success rate was calculated as follows:

*VM success rate (%) = (number of successful VM attempts / total number of VM attempts per participant) × 100*

#### 3) IASE

IASE during VM release was measured as an exploratory physiological indicator of septal movement during VM performance [27]. Although adequate leftward septal displacement was considered in the binary clinical judgement of VM success, the measured excursion value was analysed separately to support the interpretation of VM quality and was not used as a diagnostic criterion for PFO.

#### 4) Patient discomfort

##### (1) Nurse-observed discomfort

Nurse-observed discomfort was assessed using a four-item observational scale originally developed by McLachlan [28] and modified by Seo [29], including sweating, facial expression, body movement, and vocalisation. Each item was rated on a 5-point scale, with total scores ranging from 4 to 20; higher scores indicate greater discomfort. Inter-rater reliability was reported as a weighted kappa coefficient of .73 in the original study [29]. In the present study, nurse-observed discomfort was assessed by two trained echocardiography nurses who were not involved in delivering the intervention and were not informed of participants’ group allocation. To minimize observer bias, the nurses used the same structured observation form and recorded the scores immediately during the TEE procedure. Inter-rater reliability improved from moderate after the first training session (intraclass correlation coefficient [ICC] = .60) to acceptable after the second training session (ICC = .80), following review of discrepancies and additional calibration of scoring criteria. Outcomes were recorded immediately using the same structured measurement form to minimize assessor-related bias.

##### (2) Patient-reported discomfort

Patient-reported discomfort was measured using a Numeric Rating Scale, ranging from 0 (“no discomfort”) to 10 (“worst possible discomfort”).

### 4. Intervention

#### 1) Intervention group

**Figure 2.**
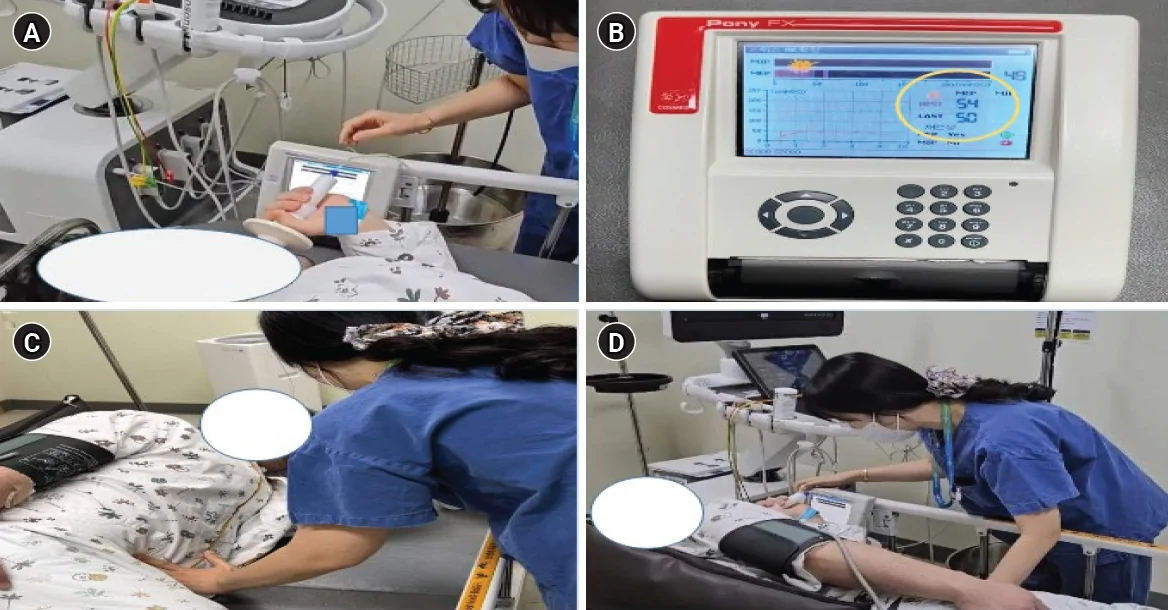
Nurse-led preprocedural Valsalva maneuver training using a maximal expiratory pressure device. (A) The patient holds the disposable mouthpiece tightly and exhales forcefully into the device while following standardized verbal instructions: “Seal your lips around the mouthpiece, exhale forcefully as if straining during defecation, and maintain the target pressure while watching the monitor.” (B) Adequate Valsalva maneuver performance was defined as maintaining a mouth pressure of approximately 54 cmH₂O (≈ 40 mmHg) for 10–15 seconds without air leakage under real-time visual feedback and nurse supervision. (C) The nurse confirms abdominal muscle contraction by applying gentle hand pressure over the lower abdomen and provides corrective verbal feedback when necessary. (D) After exhalation, the patient releases abdominal strain, completing the Valsalva maneuver cycle.

Participants in the intervention group received nurse-led preprocedural VM training using a MEP device (Pony FX, Cosmed, Rome, Italy) in two sessions: once within 1–14 days before TEE and again on the day of the procedure (Table 1). Training was conducted using standardized verbal instructions. Participants were instructed to assume the left lateral position, similar to the TEE examination posture, and to seal their lips tightly around the disposable mouthpiece to prevent air leakage. While viewing the LCD monitor of the MEP device, they were instructed to exhale forcefully as if straining during defecation and to maintain the target pressure for 10–15 seconds. Patients were trained to maintain a mouth pressure of approximately 54 cmH₂O (≈ 40 mmHg) under real-time visual feedback from the MEP device [30]. Adequate VM performance was defined as maintaining the target pressure for the required duration without air leakage. The nurse confirmed abdominal muscle contraction during VM performance and provided corrective verbal feedback when necessary (Figure 2).

The intervention was delivered by one researcher with more than 10 years of clinical experience in an echocardiography laboratory and experience in patient education related to TEE procedures and the VM. To ensure standardized intervention, the researcher used a standardized educational script and step-by-step training procedure and repeatedly checked the intervention procedure using the MEP device before participant enrollment. The intervention provider was aware of group allocation because different procedures were delivered to the intervention and control groups.

Participants were monitored for intervention-related adverse events, including dizziness, dyspnoea, chest discomfort, excessive fatigue, changes in blood pressure or pulse rate, and discontinuation of training due to discomfort, during and immediately after both VM training sessions. During VM training, the intervention was planned to be discontinued immediately if any adverse event occurred. No intervention-related adverse events were observed during the study period.

##### (1) First session (1–14 days before TEE):

The timing of the first session (1–14 days before TEE) was determined to allow sufficient time for patient familiarisation and repeated practice of the VM, while also considering the institutional scheduling system. Participants viewed a brief (4-minute) instructional video explaining the purpose of VM during TEE, correct use of the MEP device, VM training procedures, and safety precautions. After the video, supervised practice using the MEP device was performed individually. A one minute rest interval was provided between attempts, and blood pressure, pulse rate, and respiratory status were monitored to ensure patient safety.

##### (2) Second session (day of TEE):

On the day of TEE, a brief verbal review of the VM technique and MEP device use was provided immediately before the examination. Participants again practiced VM while viewing the LCD monitor and maintaining the target pressure. To minimise fatigue before the procedure, VM practice using the MEP device was limited to two attempts.

#### 2) Control group

Participants in the control group received routine preprocedural education currently provided in the echocardiography laboratory immediately before TEE. The education was delivered individually by the researcher using written educational materials and standardized verbal instructions and lasted approximately 10 minutes, including a question-and-answer session. The written materials included information on the purpose and procedure of TEE, the role of the VM in detecting PFO, the required examination posture, and general instructions for VM performance. The verbal instructions explained that participants should assume the left lateral position, seal their lips, and exhale forcefully as if straining during defecation when instructed during the examination. The researcher briefly confirmed participants’ understanding and abdominal muscle contraction, but no structured VM training or MEP device-assisted feedback was provided.

### 5. Data collection

This study was registered with the Clinical Research Information Service (CRIS; KCT0008944) [31]. Participants in the control group were recruited from November 16, 2023, to December 28, 2023, and those in the intervention group were recruited from January 2, 2024, to April 17, 2024, using a time-lagged design. During the TEE procedure, observational discomfort scores were assessed by two echocardiography nurses according to the standardized assessment protocol. The nurses were not involved in the preprocedural MEP device-based VM training and the group allocation labels were not disclosed to them. Prior to data collection, the two nurses independently assessed nurse-observed discomfort in three pilot TEE cases. Discrepancies across domains (sweating, facial expression, body movement, and vocalisation) were identified and discussed, and consensus was reached to standardise scoring criteria and minimise inter-rater variability. Preprocedural assessments were conducted on the day of TEE in a private examination room. Research assistants collected demographic information using a structured questionnaire. For some participants who had difficulty reading the questionnaire due to the small print size, the research assistants read the items aloud and recorded their verbal responses. This assistance was provided only to facilitate reading of the printed questionnaire and was not related to communication difficulties or cognitive impairment. Outcome variables, including the number of attempts required to achieve a successful VM, VM success rate, interatrial septal excursion, and patient discomfort (nurse-observed and patient-reported), were assessed during and immediately after the TEE procedure. IASE during VM release was recorded as an exploratory physiological indicator and was not used as a diagnostic criterion. Nurse-observed discomfort was independently evaluated by the nurse assigned to each group using standardised criteria.

All TEE examinations were performed with contrast enhancement according to institutional procedures. The adequacy of VM performance during TEE was evaluated by the same cardiologist throughout the study period. The cardiologist was blinded to participants’ group allocation and used the same predefined criteria for all participants. Adequate VM was determined based on leftward displacement of the interatrial septum immediately after release, according to the cardiologist’s clinical judgement. To ensure consistency, all assessments were conducted by the same research assistants following a uniform procedure throughout the study period.

### 6. Statistical analysis

All statistical analyses were performed using SPSS version 28.0 (IBM Corp., Armonk, NY, USA). A two-tailed *p*-value < .05 was considered statistically significant. Categorical variables were presented as frequencies and percentages, and continuous variables as means and standard deviations. Baseline homogeneity between the intervention group and control group was assessed using the chi-square test.

In accordance with the TREND (Transparent Reporting of Evaluations with Nonrandomized Designs) guideline, age, sex, education level, and previous endoscopy experience were selected a priori as potential confounders because of their clinical or theoretical relevance to VM performance and procedure-related discomfort. Age and sex were included because respiratory muscle strength and MEP vary by these factors [32]. Education level was considered relevant because it may influence health literacy and understanding of preprocedural instructions [33]. Previous endoscopy experience was included as a procedure-related factor that may affect anxiety, discomfort, and cooperation during invasive diagnostic procedures [34].

Between-group differences in outcome variables, including the number of attempts required to achieve a successful VM, VM success rate, IASE as a physiological indicator of VM adequacy, and patient discomfort measured by nurse observation and patient self-report, were first analysed using independent t-tests. Cohen’s d and 95% confidence intervals (CIs) for mean differences were calculated for unadjusted between-group comparisons. Analysis of covariance (ANCOVA) was subsequently conducted for each outcome variable to adjust for the selected potential confounders, and the adjusted results were reported as F values and *p*-values.

### 7. Ethical considerations

This study was approved by the Institutional Review Board (IRB) of Pusan National University Yangsan Hospital (IRB No. 05-2023-153). Written informed consent was obtained from all participants prior to study enrolment, and participants were informed of their right to withdraw from the study at any time without penalty. All collected data were anonymised and securely stored for three years in accordance with institutional policy. The study was conducted in full compliance with the ethical principles of the Declaration of Helsinki.

In this study, TEE was a clinically scheduled diagnostic procedure received by all participants and was not the study intervention. However, because TEE may be accompanied by procedure-related discomfort, participant safety was managed according to the standard clinical protocol of the echocardiography laboratory. During the procedure, the clinical team monitored participants’ vital signs, overall condition, and procedure-related discomfort. If severe discomfort, abnormal symptoms, or haemodynamic instability occurred, the procedure could be paused or discontinued according to the physician’s clinical judgement. No serious safety-related events occurred during TEE.

## RESULTS

### 1. General characteristics of participants

A total of 131 participants were enrolled and sequentially assigned according to the time-lagged design, with 67 participants first assigned to the control group and 64 subsequently assigned to the intervention group. All participants in the control group received standard education and were included in the final analysis. In the intervention group, two participants were excluded from the final analysis because of self-removal of the probe during TEE (n = 1) and identification of an atrial septal defect (n = 1). Thus, 129 participants were included in the final analysis: 67 in the control group and 62 in the intervention group (Figure 1). The largest proportion of participants in both groups was aged 50–69 years. In the intervention group, 72.6% were male, whereas 65.7% of the control group were male. Most participants had a college-level education or higher, and the majority were married. Prior experience with upper gastrointestinal endoscopy was reported in 21.0% of the intervention group and 17.9% of the control group. No significant differences were observed in baseline characteristics between the two groups, indicating baseline homogeneity (Table 2).

### 2. Hypothesis testing

As shown in Table 3, the results of hypothesis testing were as follows.

**Hypothesis 1.** The number of attempts required to achieve a successful VM was significantly lower in the intervention group than in the control group (1.15 ± 0.41 vs. 1.91 ± 0.77; t = −6.99, *p* < .001, 95% CI for the mean difference [−0.96, −0.56], Cohen’s d = 1.20). After adjusting for age, sex, education level, and previous unsedated endoscopy experience using ANCOVA, the intervention group required significantly fewer attempts to achieve a successful VM than the control group (F = 45.07, *p* < .001), supporting Hypothesis 1.

**Hypothesis 2.** The VM success rate was significantly higher in the intervention group than in the control group (92.74 ± 19.93% vs. 60.44 ± 28.25%; t = 7.14, *p* < .001, 95% CI for the mean difference [23.54, 41.06], Cohen’s d = 1.31). ANCOVA also showed that the intervention group had a significantly higher VM success rate than the control group after adjustment for covariates (F = 51.52, *p* < .001), supporting Hypothesis 2.

**Hypothesis 3.** IASE was significantly greater in the intervention group than in the control group (4.26 ± 0.99 vs. 3.12 ± 1.15; t = 6.00, *p* < .001, 95% CI for the mean difference [0.76, 1.51], Cohen’s d = 1.08). ANCOVA also showed significantly greater IASE in the intervention group after adjustment for covariates (F = 32.46, *p* < .001), supporting Hypothesis 3.

**Hypothesis 4.** This hypothesis was partially supported. Nurse-observed discomfort was significantly lower in the intervention group than in the control group, whereas patient-reported discomfort did not differ significantly between the two groups.

**Sub-hypothesis 4-1.** Nurse-observed discomfort was significantly lower in the intervention group than in the control group (8.56 ± 2.27 vs. 10.70 ± 2.61; t = −4.95, *p* < .001, 95% CI for the mean difference [−3.00, −1.28], Cohen’s d = 0.87). ANCOVA also showed significantly lower nurse-observed discomfort in the intervention group after adjustment for covariates (F = 21.73, *p* < .001), supporting Sub-hypothesis 4-1.

**Sub-hypothesis 4-2.** Patient-reported discomfort did not differ significantly between the intervention and control groups (6.34 ± 2.25 vs. 6.37 ± 2.41; t = −0.08, *p* = .933, 95% CI for the mean difference [−0.85, 0.79], Cohen’s d = 0.01). ANCOVA also showed no significant between-group difference after adjustment for covariates (F = 0.15, *p* = .701). Therefore, Sub-hypothesis 4-2 was not supported.

## DISCUSSION

This quasi-experimental study evaluated the effects of nurse-led VM training using a MEP device on VM performance and patient discomfort during TEE. The VM is an essential step for detecting PFO during TEE; however, it is often not adequately performed because of patients’ limited cooperation and difficulty in execution. Therefore, this study aimed to improve VM performance and enhance procedural efficiency through preprocedural training. The results showed that the intervention group required fewer attempts to achieve a successful VM, demonstrated higher VM success rates and greater interatrial septal excursion, and reported lower nurse-observed discomfort compared to the control group. These findings suggest that preprocedural VM training using visual feedback may improve procedural performance and reduce patient discomfort.

In interpreting these findings, potential confounding by baseline characteristics should be considered. Age, sex, education level, and previous endoscopy experience were selected a priori as clinically or theoretically relevant covariates, because these factors may influence respiratory performance, understanding of preprocedural instructions, anxiety, cooperation, or procedural discomfort [32-34]. The intervention effects remained significant after ANCOVA adjustment for these variables, supporting the robustness of the findings. However, because this study used a nonequivalent control group quasi-experimental design, residual confounding cannot be completely excluded.

Performing the VM during TEE is challenging because patients must generate and maintain sufficient expiratory pressure while tolerating discomfort caused by the TEE probe [10]. The difference between the control and intervention groups may be explained by the mode of preprocedural preparation. The control group received standard written education and verbal instructions, which may have been sufficient to convey procedural information but insufficient to help participants reproduce the target expiratory pressure during TEE. In contrast, the intervention group received repeated nurse-led VM training using the MEP device, which provided real-time visual feedback on expiratory pressure. This feedback may have helped participants recognize performance errors, maintain the target pressure, and develop a more concrete sense of successful VM performance [35]. In addition, repeated practice before the procedure may have reduced uncertainty regarding VM performance and increased participants’ confidence in performing the VM during TEE [36]. These mechanisms may partially explain the higher VM success rate, fewer required attempts, greater interatrial septal excursion, and lower nurse-observed discomfort observed in the intervention group.

The number of attempts required to achieve a successful VM was significantly lower in the intervention group than in the control group. In this study, the number of attempts was used as an outcome variable instead of total VM duration because time-based measures may not adequately reflect performance when patients fail to execute the maneuver correctly or discontinue prematurely. This finding is consistent with previous evidence suggesting that preprocedural education or feedback-based preparation can improve procedural performance and efficiency [37]. Furthermore, repeated VM attempts may impose physiological burden, such as changes in blood pressure and heart rate, indicating that a reduction in attempts is clinically meaningful. Therefore, adequate preprocedural VM training may help reduce unnecessary repeated attempts during the procedure.

The VM success rate was significantly higher in the intervention group than in the control group. This finding suggests that MEP device–based training may help translate verbal instruction into actual performance by allowing participants to practice the required pressure and duration before TEE. Unlike conventional education that relies mainly on verbal instruction and subjective confirmation,pressure-guided MEP device–based training enables both patients and nurses to monitor target pressure and duration in real time, potentially facilitating more accurate performance [38]. In addition, preprocedural information and training may reduce patient anxiety and enhance cooperation, contributing to improved VM success rates [39]. These findings are consistent with previous studies showing that pressure-guided or feedback-based training improves VM performance [40]. Therefore, nurse-led preprocedural VM training using an MEP device may be a useful strategy to enhance procedural efficiency by improving patients’ ability to perform the VM successfully during TEE.

IASE was greater in the intervention group, suggesting that the intervention may have improved the physiological adequacy and consistency of VM performance. This finding may reflect improved consistency of VM performance following training. Visual feedback provided by the MEP device may have helped patients recognise and maintain target pressure, thereby improving the accuracy and reproducibility of performance [12]. Thus, changes in IASE should be interpreted as a supportive indicator reflecting improved performance quality rather than as a diagnostic measure [41]. However, because IASE was included as an exploratory variable, its clinical implications should be interpreted with caution, and further studies are needed to clarify its significance.

Nurse-observed discomfort was lower in the intervention group, whereas patient-reported discomfort did not differ significantly between groups. Endoscopic procedures such as TEE are known to induce moderate to severe discomfort in many patients [42], and such discomfort may be influenced by both physical stimuli and emotional factors, resulting in individual variability. Nurse-observed discomfort is based on observable behaviors, such as facial expression and body movement, whereas patient-reported discomfort reflects subjective experiences, including pain perception, anxiety, and individual tolerance. Previous studies have reported discrepancies between observer-based and self-reported assessments [43], suggesting that these measures capture different aspects of discomfort. Therefore, lower nurse-observed discomfort in this study should not be interpreted as indicating reduced perceived discomfort. Rather, the two measures should be considered complementary, indicating that the intervention may have reduced observable behavioral signs of discomfort without significantly changing participants’ subjective discomfort experience.

Several limitations should be considered when interpreting these findings. Although the time-lagged quasi-experimental design reduced the possibility of cross-group contamination, residual confounding cannot be completely excluded. Age, sex, education level, and previous endoscopy experience were adjusted for in the ANCOVA models; however, unmeasured factors such as anxiety, motivation, or individual tolerance of TEE-related discomfort may have influenced the outcomes. Furthermore, although 64 participants were initially enrolled in the intervention group, two participants dropped out during the study, resulting in 62 participants being included in the final analysis. Consequently, the intervention group did not reach the minimum required sample size of 64 participants estimated through the a priori power analysis, and this should be considered when interpreting the study findings. In addition, although inter-rater reliability for nurse-observed discomfort reached an acceptable level after assessor training (ICC = .80), the possibility of observer bias cannot be fully excluded because the study was conducted in the same echocardiography laboratory setting. Finally, the interval between the first training session and TEE varied from 1 to 14 days depending on the institutional scheduling system, which may have influenced retention of VM performance.

Despite these limitations, the findings suggest that nurse-led preprocedural VM training using an MEP device may improve VM performance during TEE and reduce observable signs of discomfort. This intervention may be a practical and feasible nursing strategy to enhance procedural performance in clinical settings.

## CONCLUSION

This quasi-experimental study investigated the effects of nurse-led preprocedural VM training using a MEP device on VM performance and patient discomfort during TEE. The findings showed that the intervention group required fewer attempts to achieve a successful VM, had a higher success rate, and demonstrated greater IASE than the control group. Nurse-observed discomfort was also lower in the intervention group, although patient-reported discomfort did not differ significantly between groups.

These findings suggest that preprocedural VM training with real-time visual feedback may improve patients’ ability to perform an adequate VM during TEE and may enhance procedural efficiency. As a practical nurse-led intervention, MEP device–based training can be considered a feasible strategy to support patient cooperation and improve the quality of VM performance in clinical settings. Further studies using randomized controlled designs are needed to confirm the effectiveness of this intervention and to examine its applicability in patients undergoing TEE for PFO evaluation.

## Figures and Tables

**Table 1.** Structure of the Nurse-led VM Training Intervention

Session	Timing	Intervention components	Duration
First	1–14 days before TEE	Video education and MEP-guided VM training (target pressure ≈ 40 mmHg for 10–15 seconds, up to 5 attempts)	20 minutes
Second	Day of TEE	Verbal instruction and MEP-guided VM training (target pressure ≈ 40 mmHg for 10–15 seconds, up to 2 attempts)	5–10 minutes

VM = Valsalva maneuver; TEE = Transesophageal echocardiography; MEP = Maximal expiratory pressure.

**Table 2.** Homogeneity of General Characteristics Between the Two Groups (N = 129)

Characteristics	Categories	Intervention (n = 62)	Control (n = 67)	χ² (*p*)
Age (years)	≤ 49	10 (16.1)	8 (11.9)	1.22 (.542)
	50–69	35 (56.5)	35 (52.2)	
	70–80	17 (27.4)	24 (35.8)	
Sex	Men	45 (72.6)	44 (65.7)	0.72 (.397)
	Women	17 (27.4)	23 (34.3)	
Education	≤ High school	19 (30.6)	26 (38.8)	3.32 (.068)
	≥ College	43 (69.4)	41 (61.2)	
Occupation	No	35 (56.5)	39 (58.2)	0.04 (.840)
	Yes	27 (43.5)	28 (41.8)	
Marital status	Married	48 (77.4)	56 (83.6)	0.89 (.641)
	Single	6 (9.7)	6 (9.0)	
	Other	8 (12.9)	5 (7.5)	
Unsedated endoscopy experience	No	49 (79.0)	55 (82.1)	0.19 (.661)
	Yes	13 (21.0)	12 (17.9)	

Values are presented as n (%).

**Table 3.** Unadjusted and Adjusted Effects of the Intervention on Valsalva Maneuver Performance and Related Outcomes (N = 129)

Outcome	Intervention group (n = 62)	Control group (n = 67)	Mean difference [95% CI]	Cohen’s d	t (*p*)	F^†^ (*p*)
VM attempts	1.15 ± 0.41	1.91 ± 0.77	−0.76 [−0.96, −0.56]	1.20	−6.99 (< .001)	45.07 (< .001)
VM success rate (%)	92.74 ± 19.93	60.44 ± 28.25	32.30 [23.54, 41.06]	1.31	7.14 (< .001)	51.52 (< .001)
IASE (mm)	4.26 ± 0.99	3.12 ± 1.15	1.14 [0.76, 1.51]	1.08	6.00 (< .001)	32.46 (< .001)
Nurse-observed discomfort	8.56 ± 2.27	10.70 ± 2.61	−2.14 [−3.00, −1.28]	0.87	−4.95 (< .001)	21.73 (< .001)
Patient-reported discomfort	6.34 ± 2.25	6.37 ± 2.41	−0.03 [−0.85, 0.79]	0.01	−0.08 (.933)	0.15 (.701)

Values are presented as mean ± standard deviation. CI = Confidence interval; VM = Valsalva maneuver; IASE = Interatrial septal excursion.

† Adjusted for age, sex, education level, and previous unsedated endoscopy experience.
